# Proenkephalin and prognosis in heart failure with preserved ejection fraction: a GREAT network study

**DOI:** 10.1007/s00392-019-01424-y

**Published:** 2019-02-14

**Authors:** Prathap Kanagala, Iain B. Squire, Donald J. L. Jones, Thong Huy Cao, Daniel C. S. Chan, Gerry McCann, Jatinderpal K. Sandhu, Paulene A. Quinn, John McAdam, Anna-Marie Marsh, Joan E. Davies, Joachim Struck, Andreas Bergmann, Zaid Sabti, Raphael Twerenbold, Thomas Herrmann, Nikola Kozhuharov, Christian Mueller, Leong L. Ng, On behalf of the GREAT (Global REsearch on Acute Conditions Team) Network

**Affiliations:** 1 Department of Cardiovascular Sciences, Clinical Sciences Wing, NIHR Leicester Biomedical Research Centre, Glenfield Hospital, University of Leicester, Leicester, LE3 9QP UK; 2 Department of Cancer Studies, Leicester Royal Infirmary, University of Leicester, Leicester, LE1 5WW UK; 3 Sphingotec, GmbH, Hennigsdorf, Germany; 4 Department of Cardiology, Cardiovascular Research Institute Basel (CRIB), University Hospital Basel, Basel, Switzerland

**Keywords:** Heart failure, Preserved ejection fraction, Renal function, B-type natriuretic peptide, Proenkephalin, Opioids

## Abstract

**Background:**

Proenkephalin (PENK), a stable endogenous opioid biomarker related to renal function, has prognostic utility in acute and chronic heart failure. We investigated the prognostic utility of PENK in heart failure with preserved ejection fraction (HFpEF), and its relationship to renal function, Body Mass Index (BMI), and imaging measures of diastolic dysfunction.

**Methods:**

In this multicentre study, PENK was measured in 522 HFpEF patients (ejection fraction > 50%, 253 male, mean age 76.13 ± 10.73 years) and compared to 47 age and sex-matched controls. The primary endpoint was 2-years composite of all-cause mortality and/or heart failure rehospitalisation (HF). A subset (*n* = 163) received detailed imaging studies.

**Results:**

PENK levels were raised in HFpEF (median [interquartile range] 88.9 [62.1–132.0]) compared to normal controls (56.3 [47.9–70.5]). PENK was correlated to urea, eGFR, Body Mass Index and *E*/*e*′ (*r*_s_ 0.635, − 0.741, − 0.275, 0.476, respectively, *p* < 0.0005). During 2 years follow-up 144 patients died and 220 had death/HF endpoints. Multivariable Cox regression models showed PENK independently predicted 2 year death/HF [hazard ratio (for 1 SD increment of log-transformed biomarker) HR 1.45 [95% CI 1.12–1.88, *p* = 0.005]], even after adjustment for troponin (HR 1.59 [1.14–2.20, *p* = 0.006]), and Body Mass Index (HR 1.63 [1.13–2.33, *p* = 0.009]). PENK showed no interaction with ejection fraction status for prediction of poor outcomes. Net reclassification analyses showed PENK significantly improved classification of death/HF outcomes for multivariable models containing natriuretic peptide, troponin and Body Mass Index (*p* < 0.05 for all).

**Conclusions:**

In HFpEF, PENK levels are related to BMI, and measures of diastolic dysfunction and are prognostic for all-cause mortality and heart failure rehospitalisation.

**Electronic supplementary material:**

The online version of this article (10.1007/s00392-019-01424-y) contains supplementary material, which is available to authorized users.

## Introduction

Heart failure prognosis remains poor, with recent studies suggesting a shift in presentations from heart failure with reduced ejection fraction (HFrEF) to those with preserved ejection fraction (HFpEF) [1]. The survival of patients with HFpEF is slightly better than those with HFrEF [2] but has remained unchanged, with improvements seen only in HFrEF due to advances in therapies. Many biomarkers for prognosis in HFrEF exist, but there are scarce data in HFpEF [3] apart from natriuretic peptides [4]. There is therefore an unmet clinical need for biomarkers in HFpEF that may have a role in patient monitoring [5].

Recent work has suggested a role for the endogenous opioid system (in particular enkephalins) in determining prognosis in a variety of cardiovascular and emergency presentations, including acute myocardial infarction, acute and chronic heart failure (predominantly HFrEF), and stroke [6–9]. PENK levels may detect worsening renal function before a rise in plasma creatinine [9]. In cardiac surgery, PENK also predicts acute kidney injury [10]. The cardio-depressor effect of enkephalins on blood pressure and heart rate may play a role in the poor outcomes of these patients, as reviewed recently [11]. In contrast, enkephalins may also be a counter-regulatory mechanism (e.g. through the delta receptor which is highly expressed in kidney tissue [12], enkephalins may alter renal blood flow, increase diuresis and natriuresis, and could also be cardioprotective [11]). In all these studies, a stable peptide precursor of PENK [13] has been investigated since the enkephalins are unstable in plasma.

In HFpEF, kidney dysfunction is related to mortality [14]. Due to the links of enkephalins with cardiorenal status and existing evidence documenting the relationship of PENK and prognosis of cardiovascular disease, we examined the role of PENK in HFpEF. Previous studies on biomarkers in HFpEF have used a variety of cutoff values for left ventricular ejection fraction (LVEF) varying between 40 and 50%, but more recent guidelines have defined HFrEF and HFpEF as patients having ejection fractions under 40% and over 50%, respectively [15]. In the present study, we have used these ejection fraction cutoff values for patient recruitment, and investigated the relationship of the enkephalin system and renal function to prognosis in HFpEF. We have also explored the link of PENK to echocardiographic and cardiac MRI indices of diastolic dysfunction.

## Methods

### Study population

Two cohorts of HFpEF patients were recruited in university hospitals in Leicester, UK and Basel, Switzerland between February 2006 and August 2011, with 73% participating as in-patients and the rest as out-patients. The patients were acutely decompensated chronic HF patients or acute HF patients. Cardiac decompensation was defined as hospitalisation with HF as the primary reason, requiring treatment with diuretics, intravenous inotropes or nitrates. All patients had clinical or radiographic evidence of HF, age ≥ 18 years, left ventricular ejection fraction (LVEF) ≥ 50% on transthoracic echocardiography (TTE), elevated natriuretic peptide levels and/or structural heart disease such as left ventricular hypertrophy or left atrial enlargement, to define HFpEF [15].

For comparison with HF, 47 healthy asymptomatic controls (age and sex-matched) were also recruited. We included hypertensive controls (*n* = 22) as a major proportion of HFpEF patients had hypertension. We also compared the findings in HFpEF to 667 patients with HFrEF who had echocardiography studies showing LVEF < 40% (part of a cohort reported previously [9]). The exclusion criteria were: known myocardial infarction in the preceding 6 months, suspected or confirmed cardiomyopathy or constrictive pericarditis, non-cardiovascular life expectancy < 6 months, and being on renal replacement therapy. This study complied with the declaration of Helsinki and ethics approval was granted from respective research ethics committees. All patients provided written informed consent.

### Plasma sampling

Venous blood was withdrawn from recumbent patients and collected in pre-chilled tubes containing EDTA to prepare plasma. Plasma was stored at − 80 °C until analysis in a single batch.

### Echocardiography and magnetic resonance imaging

Transthoracic echocardiography was performed using standard techniques [16] and left ventricular ejection fraction (LVEF) was calculated using the biplane method of discs formula. Based on doppler mitral inflow, the following measurements were derived: peak of early filling (*E* velocity), peak of late atrial filling (*A* velocity) and *E*/*A* ratio.

In a subset of 163 patients, tissue Doppler measurements were available, and *E*/*e*′ was calculated from the mitral inflow velocity of the *E* wave, divided by the mitral annular velocity. Patients with prosthetic valves or valve calcification were not studied. The medial and lateral *E*/*e*′ values were averaged and used for analysis. In 108 patients, MRI scanning was undertaken and analysed as previously described [17]. The biplane area-length method (excluding the appendage and pulmonary veins) was employed for LA volumetric analysis [18].

### PENK assay

A modified version of the original assay for a stable peptide derived from preproenkephalin A (amino acids 119–159 of proenkephalin A, molecular weight 4586 Da) was used [8, 13]. A mouse monoclonal antibody against this peptide was used as the capture antibody, and another mouse monoclonal antibody labelled with methyl-acridinium ester was used as the detector, with bound chemiluminescence measured. In plasma from normal controls, PENK values were mean ± SEM 46.6 ± 14.1 pmol/L, with a median [range] of 45 [9–518] pmol/L.

### Other biomarker assays

In Leicester, the Centaur cTnI Ultra immunoassay (Siemens Healthcare Diagnostics) was used to measure troponin I (with 99th percentile of 0.04 µg/L). In Basel, plasma hsTnT (Roche Elecsys Assay; Roche Diagnostics GmbH, Mannheim, Germany) was measured (with 99th percentile of 0.014 µg/L).

For the natriuretic peptides, plasma NTproBNP was quantified using a sandwich immunoassay in Leicester, as described previously [19]. The Roche Elecsys NTproBNP assay was used in Basel. To normalise data from different assays, we log-transformed the natriuretic peptide and troponin values and calculated the Z transform for each site (dividing by 1 SD) before combining these values for analysis.

### Outcomes

All patients had a minimum follow-up of at least 2 years. The primary endpoint was all-cause mortality or heart failure (HF) rehospitalisation within 2 years. HF rehospitalisation was defined as a hospital readmission for which HF was the primary reason, requiring treatment with diuretics, intravenous inotropes or nitrates. Endpoints were obtained from hospital records and electronic databases. Secondary endpoints included all-cause mortality at 2 years. In cases with multiple events, the time to first event was used as the censored outcome.

### Statistical analysis

Statistical analyses were performed on SPSS Version 24 (SPSS Inc, Chicago, IL) and Stata 14 (TX, USA). Assuming an event rate of 40% at 2 years, a sample size of 500 patients would be powered (93.5% at *p* < 0.05) to detect a hazard ratio of the biomarker of 1.4, using the command stpower cox in Stata 14. Normality of distribution was assessed by visual inspection of plotted histograms and *QQ* plots and by the Kolmogorov–Smirnov test. Normally and non-Gaussian distributed variables were reported as mean (SD) or median (interquartile range), respectively. All biomarker levels were log_10_ transformed and normalised to 1 SD increment.

Normalised data were analysed using ANOVA and general linear models, and *p* values were Bonferroni-corrected for multiple comparisons. Non-Gaussian data and categorical variables were analysed using non-parametric tests [Mann–Whitney *U* test, Kruskal–Wallis test and Spearman (*r*_s_) correlations] and *χ*^2^ tests, respectively. Variables and factors that independently predict PENK levels were investigated using general linear models bootstrapped 1000 times. All models were based on cases with complete data.

Cox survival analysis was used to define a base model, including variables associated (at *p* < 0.10) with the primary composite outcome of death and/or HF and the secondary outcome of death. Hazard ratios refer to unit changes in age (years), eGFR (mL/min 1.73 m^2^), BP (mmHg), and heart rate (beats/min). *Z*-transformed natriuretic peptide or PENK levels were added to the base model to assess their prognostic performance, so that hazard ratios are normalised to 1SD increment of the log-transformed biomarkers.

Kaplan–Meier survival analysis was used to visualise cumulative survival and significance between tertiles tested using log rank tests. The probabilities of outcomes derived from logistic regression analysis were used in reclassification analysis using category-free net reclassification improvement (NRI) as described by Pencina et al. [20] to assess biomarker performance in up- or down-classifying risk in those with and without endpoints at 2 years.

## Results

### Patient characteristics

Patient characteristics from the two centres are reported in Supplementary Table 1. Basel patients were on average older and had lower Body Mass Index (BMI), with higher prevalences of ischemic heart disease, renal failure, hypertension, chronic obstructive pulmonary disease (COPD) and peripheral vascular disease (PVD) and less diabetes. Plasma urea, creatinine and PENK were higher in Basel patients, although eGFR was similar in both centres. Leicester patients received less β-blockers and more aldosterone receptor antagonists.

Patients with HFpEF were compared to normal controls without HF. Differences in age, sex distribution, and Body Mass Index are reported in Supplementary Table 2. HFpEF patients also had elevated *E*/*e*′ and PENK levels compared to normal controls (*p* < 0.0005). Patients with HFpEF were older, had higher BMI and with a female preponderance compared to HFrEF patients. HFrEF patients had a higher proportion of ischaemic heart disease aetiology and lower hypertension prevalence, and had poorer renal function.

HFpEF patient characteristics stratified according to PENK tertiles are reported in Table 1. Patients in the highest PENK tertile were older and had lower BMI, and had greater prevalence of ischemic heart disease, renal failure, hypertension and lower prevalence of AF. Renal dysfunction and higher NTproBNP levels were evident in the highest PENK tertile. There were no therapy differences between PENK tertiles, apart from aspirin, which may reflect the differing prevalence of ischemic heart disease. Death and the composite endpoint of death /HF were also more prevalent in the highest PENK tertile.

**Table 1:** Clinical characteristics of HFpEF patients

	All	1 < 68.2 pmol/L	2 68.2-111.6 pmol/L	3 > 111.6 pmol/L	*p* value
Number	*n* = 522	*n* = 174	*n* = 174	*n* = 174	
Demographics					
Age (years)	76.13 (10.73)	71.29 (11.50)	76.91 (9.79)	80.19 (8.82)	< 0.0005
Male (%)	253 (48.5)	91 (52.2)	83 (47.7)	79 (45.4)	0.424
Body Mass Index (kg/m^2^)	30.10 (6.89)	31.91 (7.20)	30.02 (6.15)	28.11 (6.70)	< 0.0005
Previous history					
Ischemic heart disease	171 (32.7)	41 (23.6)	63 (36.2)	67 (38.5)	0.006
Renal failure (eGFR < 90 mL/min/1.73 m^2^)	155 (29.7)	14 (8.0)	41 (23.5)	100 (57.5)	< 0.0005
Heart failure	243 (46.7)	74 (42.5)	83 (47.7)	86 (50.0)	0.361
Hypertension	425 (81.4)	130 (74.7)	147 (84.4)	148 (85.1)	0.021
Diabetes mellitus	189 (36.2)	67 (38.5)	59 (33.9)	63 (36.2)	0.672
AF	222 (42.6)	81 (46.6)	88 (50.6)	53 (30.6)	< 0.0005
Stroke	82 (15.7)	22 (12.6)	25 (14.4)	35 (20.2)	0.126
COPD	87 (16.7)	35 (20.1)	23 (13.2)	29 (16.8)	0.226
PVD	61 (11.7)	15 (8.6)	18 (10.3)	28 (16.2)	0.072
Initial observations					
Systolic BP (mmHg)	142.68 (26.70)	145.58 (25.27)	144.53 (26.16)	137.92 (28.11)	0.015
Heart rate (beats/min)	83.44 (25.03)	85.05 (25.53)	84.53 (24.78)	80.73 (24.70)	0.218
Ejection fraction (%)	58.46 (6.29)	57.93 (6.18)	58.03 (5.85)	59.43 (6.76)	0.049
NYHA class 1 (%)	46 (8.9)	27 (15.5)	17 (9.9)	2 (1.1)	< 0.0005
NYHA class 2 (%)	90 (17.4)	34 (19.5)	33 (19.2)	23 (13.5)	0.391
NYHA class 3 (%)	235 (45.5)	73 (41.9)	77 (44.3)	90 (51.7)	0.161
NYHA class 4 (%)	146 (28.2)	40 (22.9)	47 (27.3)	59 (34.5)	0.056
Plasma biomarkers					
Urea (mmol/L)	9.79 (5.51)	6.70 (2.13)	8.49 (3.24)	14.29 (6.77)	< 0.0005
Creatinine (µmol/L)	113.89 (56.81)	82.59 (21.52)	101.95 (29.59)	156.89 (73.28)	< 0.0005
eGFR (mL/min/1.73 m^2^)	59.98 (24.95)	78.33 (22.69)	61.33 (18.28)	40.39 (17.32)	< 0.0005
Haemoglobin (g/L)	123.6 (20.8)	133.3 (19.6)	123.5 (18.8)	114.0 (19.5)	< 0.0005
Troponin I (µg/L)	0.17 (0.58)	0.18 (0.59)	0.19 (0.62)	0.14 (0.52)	0.082
Sodium (mmol/L)	138.16 (5.03)	138.54 (4.68)	137.97 (5.53)	137.97 (4.84)	0.481
NTproBNP (pmol/L) Leicester	2312.6 [1128.7–4143.7]	650.6 [77.7–1817.3]	1048.6 [479.9–2205.9]	1569.6 [729.7–2876.3]	0.008
NTproBNP(pg/mL) Basel	4897.5 [2653.5–10,572.5]	1673.5 [644–3431.5]	3281.5 [1484.3–4837.5]	6365.5 [3121.5–10,827.3]	< 0.0005
Treatment					
Aspirin	194 (37.5)	48 (27.6)	75 (43.1)	71 (41.8)	0.004
β-Blocker	326 (62.9)	103 (59.2)	113 (64.9)	110 (64.7)	0.456
ACE inhibitor or ARB	390 (74.7)	134 (77.0)	136 (78.2)	120 (69.0)	0.099
Aldosterone antagonists	64 (12.2)	23 (13.2)	27 (15.5)	14 (8.0)	0.094
Statin	183 (48.3)	45 (42.1)	65 (49.6)	73 (51.8)	0.295
Digoxin	61 (11.8)	20 (11.5)	24 (13.8)	17 (10.0)	0.546
End points (2 years)					
Death	144 (27.7)	25 (14.4)	44 (25.4)	75 (43.1)	< 0.0005
Death and/or heart failure	220 (42.1)	45 (25.9)	76 (43.7)	99 (56.9)	< 0.0005

Characteristics of the 522 HFpEF patients in the Leicester and Basel cohorts, according to PENK tertiles. NTproBNP is reported for the cohorts, respectively. Body Mass Index measurements were available in 411 cases. Numerical data are presented as *n* (%) and mean (SD) or median (Interquartile range) are reported. *p* values are quoted for the ANOVA/Kruskal Wallis or Chi squared tests for continuous or categorical variables, respectively

*ARB* angiotensin 2 receptor blocker

### Correlation analysis

PENK was correlated to age (*r*_s_ 0.363, *p* < 0.0005), Body Mass Index (BMI) (*r*_s_ − 0.275, *p* < 0.0005, *n* = 411), eGFR (− 0.741, *p* < 0.0005), plasma creatinine (0.667, *p* < 0.0005), plasma urea (0.635, *p* < 0.0005), *Z* score of log natriuretic peptides (0.437, *p* < 0.0005), heart rate (− 0.094, *p* = 0.032) and systolic BP (− 0.145, *p* = 0.001). The relationship of PENK with eGFR or BMI in HFpEF is illustrated in Supplementary Fig. 1.

Multivariable general linear modelling was used to determine variables that showed independent association with PENK. Supplementary Table 3 shows the independent significant predictors were plasma urea, eGFR, natriuretic peptide levels, age, AF, previous history of IHD, and renal failure. In the subset (*n* = 411) for which BMI was available, BMI was retained as an independent predictor with the above variables, whilst age and AF were excluded. The predictors accounted for 64–69% of the variance in PENK levels.

### Echocardiographic and magnetic resonance imaging parameters: relationship with PENK levels

**Fig. 1:**
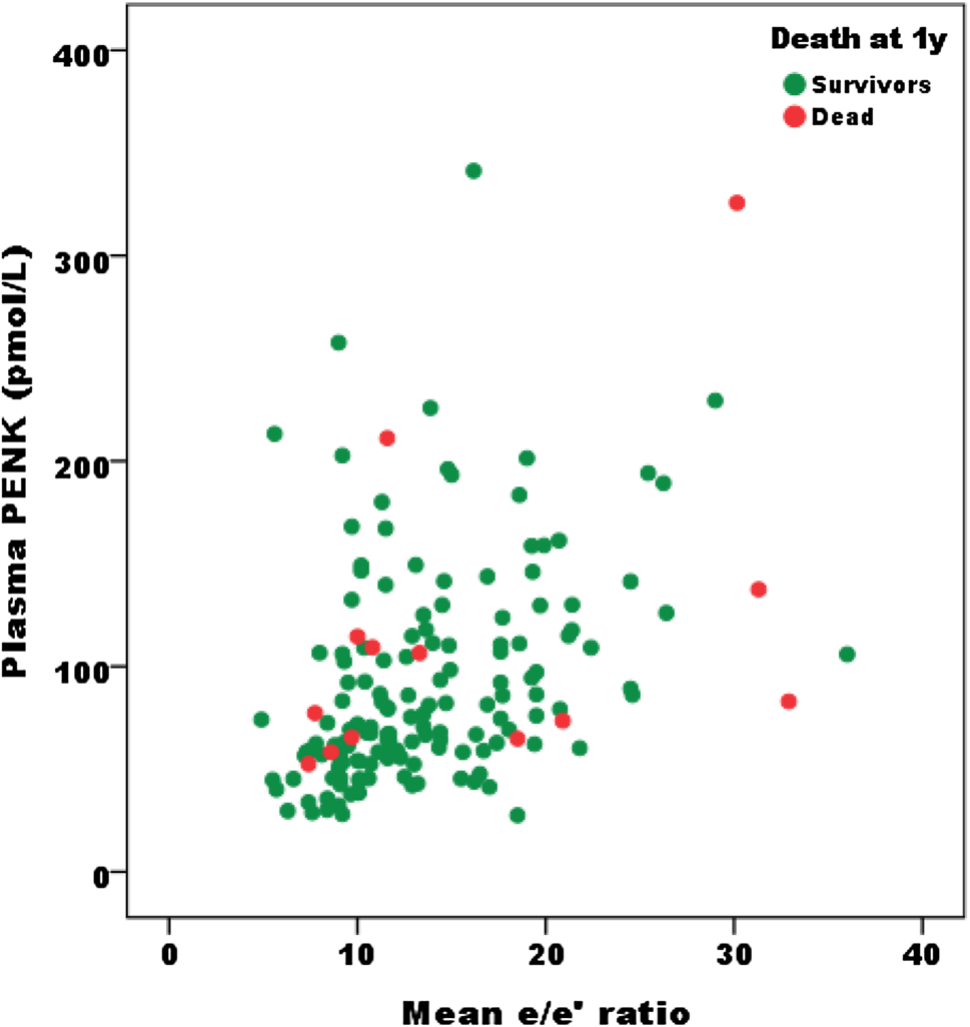
PENK relationship to the echocardiographic diastolic index *E*/*e*′. Correlation of PENK with echocardiographic index of diastolic dysfunction *E*/*e*′. The Spearman correlation coefficient was 0.453 (*p* < 0.0005)

In a subset of patients in whom tissue Doppler measurements were available, PENK was correlated to *E*/*e*′ (0.453, *p* < 0.0005, *n* = 163: Fig. 1). The mean (SD) for *E*/*e*′ according to increasing PENK tertiles was 10.67 (3.42), 14.14 (5.51), and 16.57 (6.58), respectively, [ANOVA *p* < 0.0005, with the top tertile statistically different from the 1st tertiles (Bonferroni-corrected *p* < 0.0005) and 2nd (*p* = 0.046)].

**Fig. 2:**
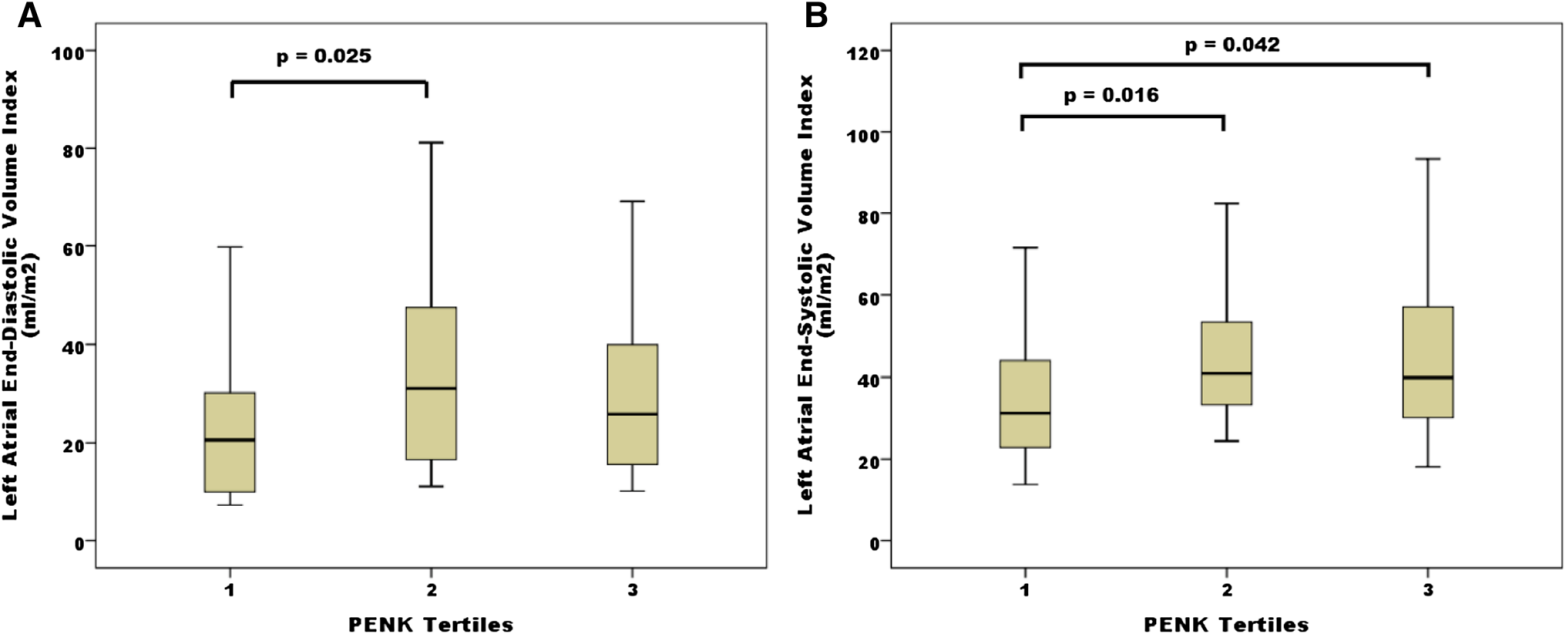
MRI-derived ventricular volumes according to PENK tertiles. Box and whisker plots of **a** LAEDVI and **b** LAESVI according to PENK tertiles. LAEDVI and LAESVI differed between PENK tertiles (ANOVA *p* = 0.024 and 0.01). Significant differences were seen between the second and first tertile of LAEDVI (*p* = 0.025) and between second and third tertiles of LAESVI and the first tertile (*p* = 0.016 and 0.042, respectively)

Cardiac magnetic resonance imaging (MRI) left atrial volume indices in systole and diastole (LAEDVI, LAESVI) were available in a subset of 108 HFpEF patients. Figure 2 shows box plots of these volumes according to PENK tertiles. LAEDVI and LAESVI differed between PENK tertiles (ANOVA *p* = 0.024 and 0.01). The second tertile of LAEDVI was higher than the first (*p* = 0.025). Both second and third tertiles of LAESVI were higher than the first (*p* = 0.016 and 0.042, respectively). The MRI derived left ventricular end diastolic and end systolic mass indices (LVEDMI and LVESMI) were similar between the PENK tertiles, as were the echocardiography-measured LV mass indices and LVEDM/LVEDV ratio (*p* non-significant).

### Survival analysis

**Fig. 3:**
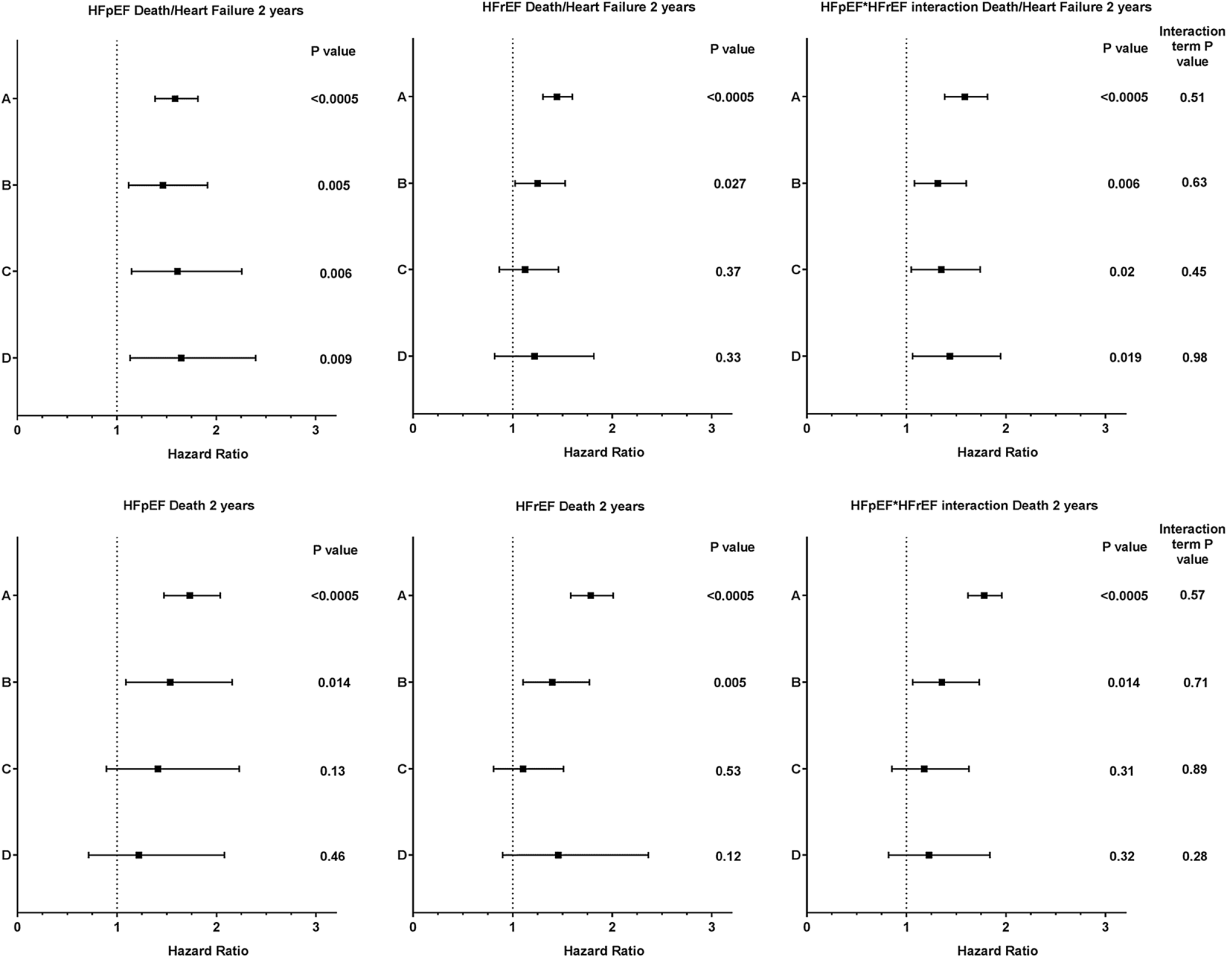
Hazard ratios for PENK in Cox survival analysis for Death/HF or Death at 2 years in HFpEF and HFrEF. Forest plots showing hazard ratios and confidence intervals for PENK (as a univariable in A) following adjustment for a multivariable base model (B, containing the variables age, gender, NYHA class IV, past history of heart failure, ischemic heart disease, hypertension, diabetes, atrial fibrillation, systolic BP, heart rate, plasma urea, creatinine, sodium, haemoglobin, natriuretic peptide), base model with troponin (C), and base model with troponin and BMI (D). Hazard ratios for interaction of PENK with ejection fraction status are shown on the right of the figure

During follow-up for 2 years, there were 144 deaths and 220 death/HF endpoints. As PENK tertile increased, so did the incidence of death alone or of the composite end-point of death or heart failure hospitalisation. Cox proportional hazard survival modelling was used to investigate factors associated with the outcome of death/HF at 2 years, and hazard ratios for PENK (for 1 SD increment of the log-transformed biomarker) are reported in Fig. 3.

The base model contained the variables age, gender, NYHA class IV, past history of heart failure, ischemic heart disease, hypertension, diabetes, atrial fibrillation, systolic BP, heart rate, plasma urea, creatinine, sodium, haemoglobin, and natriuretic peptide. This contained variables that have been related to outcome, or had univariable association with the outcome (*p* < 0.05). Univariable hazard ratios for the base model variables are reported in Supplementary Table 4. The HR for PENK (univariable HR A in Fig. 3), remained significant when adjusted for the base model (HR B in Fig. 3). The HR for PENK was significant on further adjustment with the base model and troponin (HR C in Fig. 3), and on addition of BMI to the base model with troponin (HR D in Fig. 3).

Cox survival modelling of HFrEF patients showed PENK remained significant on adjustment with the base model variables, although not following further adjustment with addition of troponin and BMI to the models. On interaction analysis in the whole population, PENK hazard ratios remained significant following adjustment using the base model, troponin, and BMI. Interaction terms of PENK with HFpEF/HFrEF status were non-significant suggesting similar performance of the biomarker irrespective of ejection fraction.

The lower panel of Fig. 3 illustrates the hazard ratios of PENK for death at 2 years (unadjusted, A) and following adjustment using the base model (B), with further additions of troponin (C) or BMI (D). PENK hazard ratios were significant following adjustment using the base model but not with further addition of troponin or BMI. Interaction analysis suggested PENK had similar performance irrespective of ejection fraction.

**Fig. 4:**
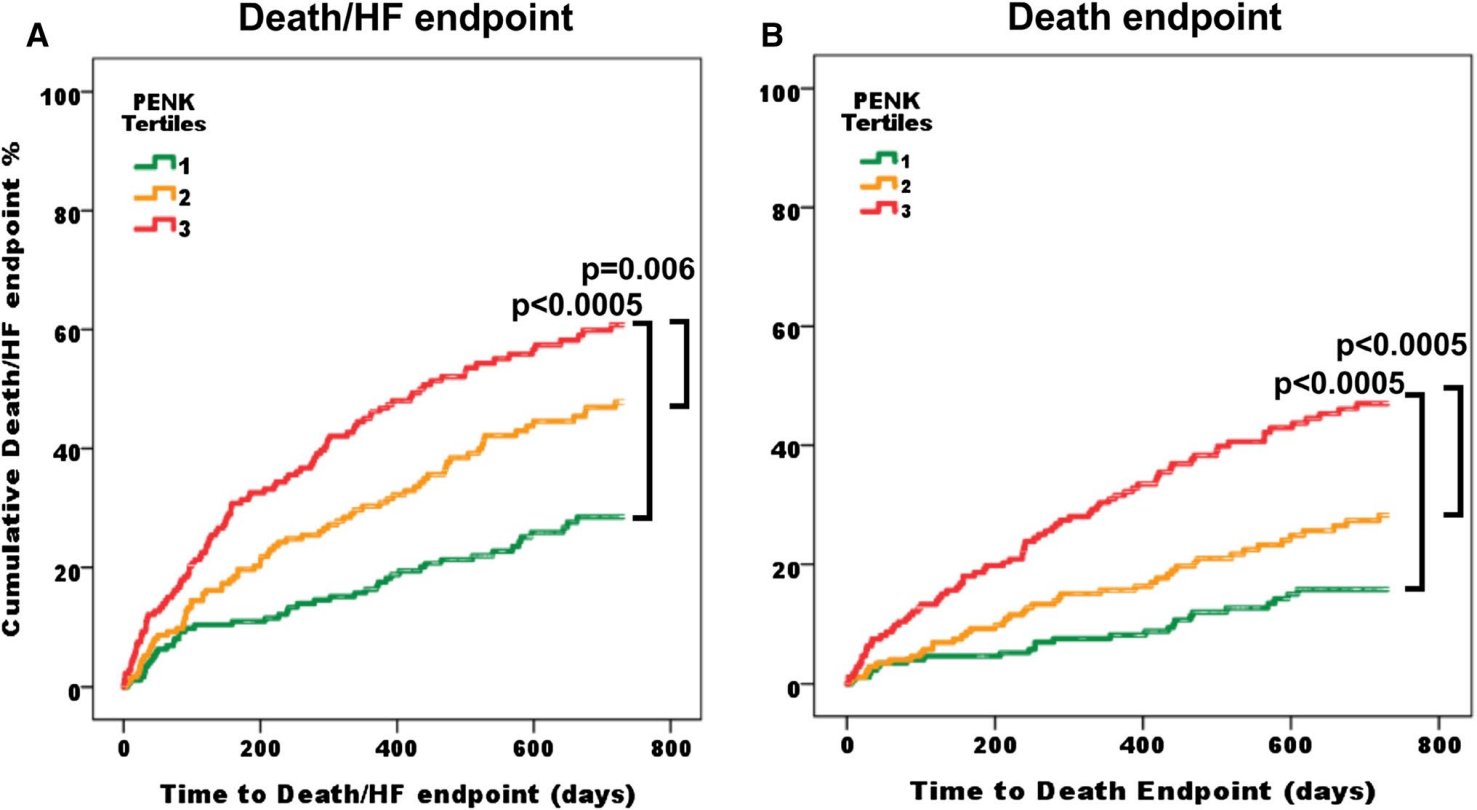
Kaplan–Meier survival analysis for death/HF hospitalisation and all-cause mortality. Kaplan–Meier plots of **a** the composite endpoint of death and/or HF hospitalization and **b** all-cause mortality, according to PENK tertiles. Log rank tests showed differences between tertiles 1 and 3 (*p* < 0.0005 for both endpoints), and between tertiles 2 and 3 (*p* = 0.006 for death/HF and *p* < 0.0005 for death)

Kaplan–Meier analysis (Fig. 4) of the HFpEF cohort showed patients in the highest tertile had worse outcomes than those in tertile 1 (*p* < 0.0005) or 2 (*p* = 0.006) for the endpoint of death/HF. For all-cause mortality, patients in the highest tertile had worse survival than those in tertile 1 (*p* < 0.0005) or 2 (*p* < 0.0005).

### Reclassification analyses and *C* statistics

Logistic regression model derived risk scores for death/HF at 2 years using base model variables with further addition of troponin and BMI, were used with addition of PENK to calculate the continuous net reclassification improvement index NRI (> 0) (Table 2). PENK showed significant net reclassification improvement on the base model, and on addition of troponin and BMI.

**Table 2:** *C* statistics and reclassification analysis for death/HF or death at 2 years using biomarkers

Model	*C* statistic (95% confidence interval)	Model + PENK *C* statistic (95% confidence interval)	*P* value	Reclassification analysis	
				NRI (95% confidence interval)	*P* value
Death/HF 2 years					
A	0.69 (0.63–0.75)				
B	0.69 (0.63–0.75)	0.70 (0.64–0.77)	NS	18.9 (0.10–37.8)	0.049
C	0.70 (0.64–0.76)	0.72 (0.66–0.78)	NS	24.4 (2.2–46.5)	0.031
D	0.70 (0.64–0.76)	0.72 (0.66–0.78)	NS	26.6 (2.7–50.5)	0.029
Death 2 years					
A	0.66 (0.61–0.75)				
B	0.79 (0.73–0.85)	0.79 (0.72–0.85)	NS	22.3 (0.50–44.0)	0.045
C	0.81 (0.75–0.87)	0.81 (0.75–0.87)	NS	11.5 (− 14.6 to 37.5)	0.38
D	0.82 (0.76–0.88)	0.82 (0.76–0.88)	NS	− 11.1 (− 40.3 to 18.0)	0.45

C statistics and reclassification analysis, for the outcomes of death/HF or death at 2 years, using continuous reclassification showing the net reclassification improvement of adding PENK to the base model, and models with troponin and BMI

A, univariable PENK *C* statistic

B, base model (containing variables age, gender, NYHA class IV, past history of heart failure, ischemic heart disease, hypertension, diabetes, atrial fibrillation, systolic BP, heart rate, plasma urea, creatinine, sodium, haemoglobin, and natriuretic peptide)

C, base model with troponin

D, base model with troponin and BMI

For the outcome of death at 2 years, PENK showed significant net reclassification improvement on the base model, but not when troponin or BMI were added to the base model. The increments in C statistic on addition of PENK to the base model, or models with troponin and BMI were not significant. Areas under the receiver operating characteristic curves for PENK, natriuretic peptides, troponin and the combination of all three for the outcomes of death/HF or death at 2 years are illustrated in Supplementary Fig. 2.

## Discussion

Although many biomarkers have been described for diagnosis or prognosis in HFrEF, few biomarkers in HFpEF perform beyond base models of clinical variables [3]. Natriuretic peptides [4] have been shown to independently predict outcomes in HFpEF. However, many previous reports were based on clinical trials, and may not have used the contemporary definition of cutoff values of ejection fraction for HFpEF (ejection fraction ≥ 50%) [15]. There is a clinical need for such biomarkers in HFpEF as they may facilitate clinical care, as well as the search for therapies that may influence outcomes.

In this study of HFpEF patients, as defined by contemporary cutoff values in ejection fraction, we have confirmed that PENK is a strong correlate of renal function, and provides prognosis for the composite outcome of death and/or HF hospitalisation. In these multivariable models, PENK emerged as a significant marker for death/HF, even following adjustment for clinical variables that have previously been reported as prognostic markers, such as AF [21] and anaemia [22]. PENK remained an independent marker for death/HF even following adjustment for troponin and Body Mass Index. The performance of PENK as a prognostic marker for death/HF was independent of ejection fraction, as there was no significant interaction with ejection fraction status (reduced or preserved). We also used reclassification analysis [20], which confirmed the prognostic performance of PENK for the composite death/HF endpoint. For the endpoint of death alone, PENK remained a significant prognostic marker following addition to the base model, but not when troponin was added to the model. However, PENK did not improve the C statistic significantly for poor outcomes when added to any of the models. It has been reported that reclassification analysis is more sensitive and less conservative when assessing utility of biomarkers in models [23].

We have previously reported PENK as a predictor of poor outcomes in acute heart failure [9] (predominantly HFrEF and HFmEF as defined in current guidelines [15]), and in acute myocardial infarction [8], and the association with worsening renal function [8]. The current findings complement these previous reports. Enkephalins have a cardiodepressor effect with a negative inotropic effect, lowered BP and reduced tissue perfusion [11, 24]. Reduced renal perfusion would be a potential mechanism by which PENK might directly influence eGFR. The current findings reinforce these previous reports of the strong association of PENK with renal function and poor outcomes, and may suggest the PENK system as a suitable target for therapeutic intervention, as well as a marker of prognosis, in HFpEF.

In a subgroup of these HFpEF patients, we have demonstrated a correlation of PENK with the tissue Doppler echocardiography parameter E/e′, a measure of the left ventricular filling pressure that reflects diastolic dysfunction. Further evidence in support of this was the larger left atrial volume indices seen in the second and third tertiles of PENK.

Body Mass Index was available in a subset of the HFpEF patients, and a negative correlation was observed with PENK levels, resembling the findings with natriuretic peptides. This relationship was independent as general linear modelling retained BMI in addition to renal function measures, natriuretic peptide levels and previous history of renal failure and ischemic heart disease. A previous study has also noted a negative relationship between PENK and weight and body surface area [6]. Enkephalins are widely distributed in many tissues [12] and have a lipolytic effect on adipose tissue [25]. A recent finding may be of relevance to this negative association of enkephalins with body weight, as group 2 innate immune cells (ILC2) may have a “beiging” effect on white adipose, converting it to higher energy expending brown fat with met-enkephalin as the likely candidate factor [26].

## Limitations

Our findings are based on two European centres recruiting heart failure patients and should be validated in additional, non-European populations. While there were clinically relevant differences in patient characteristics between the two centres, our findings are based on models corrected for these differences. Different natriuretic peptide assays were used, and we tried to mitigate this using *Z* transforms of the logged natriuretic peptide levels. Body Mass Index was not available on all patients and tissue Doppler echocardiography and MRI studies were only performed on a subgroup of patients. The patients who underwent cardiac MRI were younger and had better renal function than the rest of the cohort, since renal impairment precluded the use of MRI contrast.

## Conclusions

In HFpEF, PENK provides prognostic information on the composite outcome of all-cause mortality and/or heart failure rehospitalisation, reflecting echocardiographic measures of diastolic dysfunction. High PENK levels are associated with increased ventricular and atrial volume indices, lowered Body Mass Index and renal impairment.

## Electronic supplementary material

Below is the link to the electronic supplementary material.

Supplementary material 1 (DOCX 637 KB)

## Abbreviations

BMI: Body mass index
BNP: B-type natriuretic peptide
COPD: Chronic obstructive pulmonary disease
HF: Heart failure
HFpEF: Heart failure with preserved ejection fraction
HFrEF: Heart failure with reduced ejection fraction
LVEF: Left ventricular ejection fraction
MRI: Magnetic resonance imaging
NRI: Net reclassification improvement
NTproBNP: N-terminal pro-B-type natriuretic peptide
PENK: Amino acids 119–159 of proenkephalin A
PVD: Peripheral vascular disease
