# Supplementary material for: Proenkephalin and prognosis in heart failure with preserved ejection fraction: a GREAT network study
Source: Clin Res Cardiol. 2019 Feb 14;108(8):940–9. doi: 10.1007/s00392-019-01424-y (PMC6652170; doi:10.1007/s00392-019-01424-y)
Supplement: Supplementary file 1 — Supplementary material 1 (DOCX 637 KB) [file 392_2019_1424_MOESM1_ESM.docx]

## Supplementary Figures and Tables

**Supplementary Fig.1** **Title: Scatter plots of plasma PENK and eGFR or Body Mass index.**

The Spearman correlation coefficients were -0.741 (p<0.0005) for eGFR **(a)** and -0.275 (p<0.0005) for body mass index **(b)** respectively.

**Online Supplementary Fig.1**

**
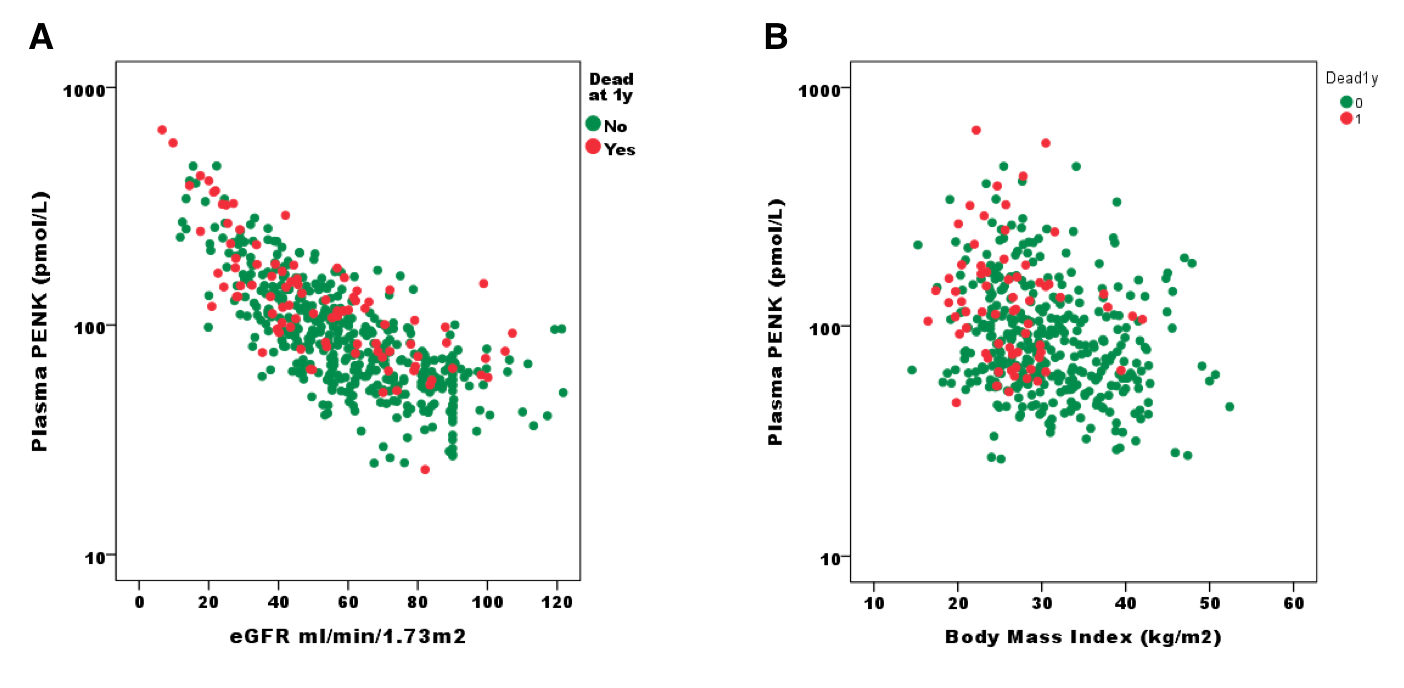
**

**Online Supplementary Fig.2 Title: Receiver Operating Characteristic curves for PENK, natriuretic peptides, troponin and the combination of all 3, for the outcomes of death/HF or death at 2 years.** Areas under the ROC curves are reported next to the biomarkers.

**
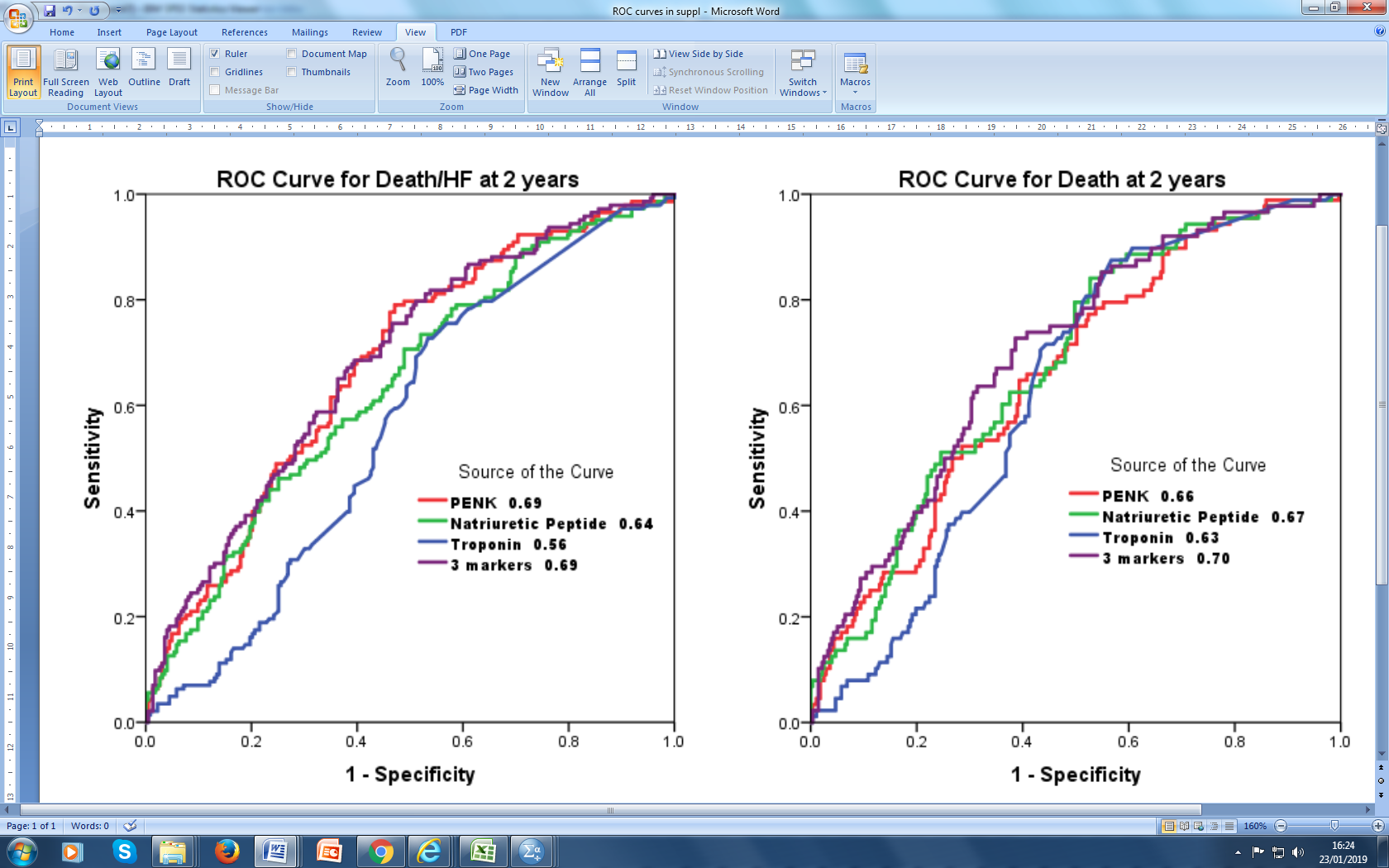
Online Supplementary Table 1: Clinical characteristics of HFpEF patients according to recruitment site.**

Characteristics of the Leicester and Basel cohorts of patients with HFpEF (EF≥50%), in patients with available ejection fraction measurements samples. Numerical data are presented as n (%) and mean (SD) or Median [Interquartile range] are reported. P values are quoted for the ANOVA/Mann Whitney or Chi squared tests for continuous or categorical variables respectively

|  | **All** | **Leicester** | **Basel** | **P Value (2 sites)** |
| --- | --- | --- | --- | --- |
| Number | 522 | 261 | 261 |  |
| **Demographics** |  |  |  |  |
| Age (years) | 76.13 (10.73) | 74.04 (10.49) | 78.22 (10.57) | <0.0009 |
| Male (%) | 253 (48.5) | 126 (48.3) | 127 (48.6) | NS |
| Body Mass index (kg m^-2^, n=411) | 30.10 (6.89) | 33.65(6.99) | 28.04 (5.93) | <0.0001 |
| **Previous History** |  |  |  |  |
| Ischemic heart disease | 171 (32.8) | 71 (27.2) | 100 (38.3) | 0.007 |
| Renal Failure | 155 (29.7) | 25 (9.6) | 130 (49.8) | <0.0005 |
| Heart Failure | 243 (46.7) | 143 (54.8) | 100 (38.6) | <0.0005 |
| Hypertension | 425 (81.4) | 202 (77.4) | 223 (85.4) | 0.018 |
| Diabetes Mellitus | 189 (36.2) | 114 (43.7) | 75 (28.7) | <0.0005 |
| Atrial Fibrillation | 222 (42.6) | 127 (48.8) | 95 (36.4) | NS |
| Stroke | 82 (15.7) | 46 (17.6) | 36 (13.8) | NS |
| COPD | 87 (16.7) | 27 (10.3) | 60 (23.1) | <0.0005 |
| PVD | 61 (11.7) | 7 (2.7) | 54 (20.8) | <0.0005 |
| **Initial Observations** |  |  |  |  |
| Heart rate (beats/min) | 83.44 (25.03) | 80.38 (23.39) | 86.47 (26.26) | NS |
| Systolic BP (mm Hg) | 142.68 (26.70) | 142.43 (25.82) | 142.93 (27.59) | NS |
| Plasma Urea (mmol/L) | 9.79 (5.51) | 8.80 (4.18) | 10.89 (6.52) | <0.0005 |
| Plasma Creatinine (μmol/L) | 113.90 (56.81) | 105.58 (39.30) | 122.38 (69.38) | 0.001 |
| Plasma Sodium (mmol/L) | 138.16 (5.03) | 138.24 (4.78) | 138.08 (5.28) | NS |
| Haemoglobin (g/L) | 123.63 (20.84) | 124,90 (21.82) | 122.35 (19.76) | NS |
| eGFR (ml/min/1.73m^2^) | 59.98 (24.95) | 61.10 (20.37) | 58.84 (28.89) | NS |
| Troponin (µg/L) | 0.17 (0.58) | 0.25 (0.75) | 0.09 (0.29) | NS |
| **Echocardiography** |  |  |  |  |
| Ejection Fraction (%) | 58.46 (6.30) | 58.20 (6.81) | 58.72 (5.76) | NS |
| E/e' (n=163) | 12.3 [9.64 – 17.4] | 12.1 [9.50-17.6] | 13.24 [10.15-15.86] | NS |
| **Treatment** |  |  |  |  |
| Aspirin | 194 (37.5) | 96 (36.8) | 98 (38.1) | NS |
| β-blocker | 326 (62.9) | 148 (56.7) | 178 (69.3) | 0.003 |
| ACE inhibitor or ARB* | 390 (74.7) | 200 (76.6) | 190 (72.8) | NS |
| Aldosterone antagonist | 64(12.3) | 64 (24.5) | 0 (0) | <0.0005 |
| Statin | 183 (48.3) | 51 (41.5) | 132 (51.6) | NS |
| Digoxin | 61 (11.8) | 49 (18.8) | 12 (4.7) | NS |
| **Biomarkers** |  |  |  |  |
| PENK (pmol/L) | 89.0 [62.1-132.0] | 81.3 [58.6-120.2] | 96.3 [63.9-146.8] | 0.002 |
| NTproBNP(pmol/L) |  | 1254 [546.9-2246.8] | - |  |
| BNP (pg/mL) |  | - | - |  |
| NTproBNP (pg/mL) |  | - | 3414 [1531.5-6748] |  |
|  |  |  |  |  |

**Online Supplementary Table 2: Comparison of HFpEF patients with Normal controls and HFrEF patients.**

Characteristics of the Leicester and Basel cohorts of patients with HFpEF (Ejection fraction ≥50%) compared to non-heart failure controls and HFrEF (ejection fraction <40%). Numerical data are presented as n (%) and mean (SD) or Median [Interquartile range] are reported. P values are quoted for the ANOVA/Mann-Whitney or Chi squared tests for continuous or categorical variables respectively and are corrected by the Bonferroni procedure for multiple comparisons.

| . | **Controls (Con)** | **HFpEF** | **P Value (HFpEF vs Con)** | **HFrEF** | **P Value (HFpEF vs HFrEF)** |
| --- | --- | --- | --- | --- | --- |
|  |  |  |  |  |  |
| Number | 47 | 522 |  | 667 |  |
| **Demographics** |  |  |  |  |  |
| Age (years) | 73.06 (5.07) | 76.13 (10.73) | NS | 73.68 (11.84) | 0.001 |
| Male (%) | 23 (48.9) | 253 (48.5) | NS | 490 (73.6) | <0.0005 |
| Body Mass index (kg m^-2^) | 25.45 (3.13) | 30.10 (6.89) | <0.0005 | 28.14 (6.14) | <0.0005 |
| **Previous History** |  |  |  |  |  |
| Ischemic heart disease | 0 (0) | 171 (32.8) | <0.0005 | 311 (46.6) | <0.0005 |
| Renal Failure | 0 (0) | 155 (29.7) | <0.0005 | 240 (36.0) | 0.021 |
| Heart Failure | 0 (0) | 243 (46.7) | <0.0005 | 329 (49.3) | NS |
| Hypertension | 22 (46.8) | 425 (81.4) | <0.0005 | 424 (63.6) | <0.0005 |
| Diabetes Mellitus | 0 (0) | 189 (36.2) | <0.0005 | 206 (30.9) | 0.053 |
| AF | 0 (0) | 222 (42.6) | <0.0005 | 273 (41.5) | NS |
| Stroke | 0 (0) | 82 (15.7) | 0.003 | 104 (15.7) | NS |
| COPD | 1 (2.1) | 87 (16.7) | 0.008 | 90 (13.5) | NS |
| PVD | 0 (0) | 61 (11.7) | 0.013 | 63 (9.6) | NS |
| **Initial Observations** |  |  |  |  |  |
| Heart rate (beats/min) | 67.46 (10.54) | 83.44 (25.03) | <0.0005 | 93.26 (25.87) | <0.0005 |
| Systolic BP (mm Hg) | 150.19 (23.79) | 142.68 (26.70) | NS | 131.55 (25.97) | <0.0005 |
| Plasma Urea (mmol/L) | 6.17 (1.50) | 9.79 (5.51) | <0.0005 | 11.30 (6.48) | <0.0005 |
| Plasma Sodium (mmol/L) | 140.38 (1.75) | 138.16 (5.03) | 0.011 | 137.8 (5.12) | NS |
| Haemoglobin (g/L) | 140.11 (14.51) | 123.63 (20.84) | <0.0005 | 126.84 (20.87) | 0.029 |
| eGFR (ml/min/1.73m^2^) | 82.79 (10.54) | 59.98 (24.95) | <0.0005 | 55.66 (24.01) | 0.007 |
| Troponin I (µg/L) | NA | 0.17 (0.58) | NA | 0.25 (2.35) | <0.0005 |
| **Echocardiography** |  |  |  |  |  |
| Ejection Fraction (%) | 57.99 (4.69) | 58.46 (6.30) | NS | 26.53 (7.49) | <0.0005 |
| E/e' (n=299) | 8.30  [6.75-10.50] | 12.30 [9.64-17.40] | <0.0005 | 15.6 [12.3 -21.0] | <0.0005 |
| **Treatment** |  |  |  |  |  |
| Aspirin | 3 (6.4) | 194 (37.5) | <0.0005 | 294 (44.4) | 0.077 |
| β-blocker | 2 (4.3) | 326 (62.9) | <0.0005 | 340 (51.4) | NS |
| ACE inhibitor or ARB* | 10 (21.3) | 390 (74.7) | <0.0005 | 414 (62.1) | NS |
| Aldosterone antagonist | 0(0) | 64 (12.2) | 0.011 | 115 (17.2) | <0.0005 |
| Statin | 16 (34.0) | 183 (48.2) | NS | 267 (49.4) | NS |
| **Biomarkers** |  |  |  |  |  |
| PENK (pmol/L) | 56.3 [47.9-70.5] | 88.9 [62.1-132.0] | <0.0005 | 98.3 [67.0-147.8] | 0.019 |

**Online Supplementary Table 3: Predictors of plasma PENK levels**

Multivariable general linear models showing independent predictors of PENK levels. Model B consists of the variables included in Model A with the addition of Body Mass Index (n=411). Models were bootstrapped 1000 times to obtain the beta coefficients and P values.

|  | Model A | |  | Model B | |
| --- | --- | --- | --- | --- | --- |
| Variable | Beta Coefficient | P value | Variable | Beta Coefficient | P value |
| Plasma urea | 0.016 | 0.002 | Plasma urea | 0.017 | 0.002 |
| eGFR | -0.003 | 0.002 | eGFR | -0.003 | 0.002 |
| Natriuretic peptide levels | 0.049 | 0.002 | Natriuretic peptide levels | 0.041 | 0.002 |
| Age | 0.002 | 0.002 | Body Mass Index | -0.005 | 0.002 |
| AF |  | NS | Past history Renal Failure |  | NS |
| Past history IHD |  | NS | Past history IHD |  | NS |
| Past history Renal Failure |  | NS | Past history Hypertension |  | NS |
| Past history Hypertension |  | NS | AF |  | NS |
| Systolic BP |  | NS | Heart Rate |  | NS |
| Heart Rate |  | NS | Age |  | NS |
|  |  |  | Systolic BP |  | NS |
|  |  |  |  |  |  |
| P<0.0005 Adjusted R^2^ 0.64 | | | P<0.0005 Adjusted R^2^ 0.69 | | |

**Online Supplementary Table 4: Univariable Hazard Ratios for outcomes at 2 years.**

Cox survival analysis for the outcomes of death/HF at 2 years and death at 2 years, Univariable hazard ratios for the base model variables are reported, with 95% confidence intervals [95% CI].

|  | Outcome of Death/HF at 2 years | |  | Outcome of Death at 2 years | |
| --- | --- | --- | --- | --- | --- |
| Variable | Hazard Ratio [95% CI] | P value | Variable | Hazard Ratio [95% CI] | P value |
| Age | 1.03 [1.02-1.05] | <0.0005 | Age | 1.05 [1.03-1.07] | <0.0005 |
| Male Gender | 1.06 [0.82-1.38] | 0.65 | Male Gender | 1.30 [0.94-1.81] | 0.11 |
| NYHA class 4 | 1.65 [1.25-2.18] | <0.0005 | NYHA class 4 | 1.95 [1.39-2.74] | <0.0005 |
| Body Mass Index | 0.96 [0.93-0.98] | 0.001 | Body Mass Index | 0.92 [0.89-0.95] | <0.0005 |
| Past history HF | 1.36 [1.04-1.77] | 0.024 | Past history HF | 1.25 [0.90-1.74] | 0.18 |
| Past history IHD | 1.71 [1.31-2.23] | <0.0005 | Past history IHD | 1.78 [1.28-2.47] | 0.001 |
| Past history Hypertension | 1.03 [0.73-1.45] | 0.88 | Past history Hypertension | 0.68 [0.47-1.00] | 0.052 |
| Past history Diabetes | 1.10 [0.84-1.45] | 0.48 | Past history Diabetes | 0.79 [0.56-1.13] | 0.20 |
| Past history AF | 0.99 [0.76-1.30] | 0.98 | Past history AF | 0.99 [0.71-1.38] | 0.96 |
| Systolic BP | 0.99 [0.98-0.99] | 0.02 | Systolic BP | 0.99 [0.98-0.99] | 0.001 |
| Heart Rate | 1.01 [1.00-1.01] | 0.37 | Heart Rate | 1.01 [1.00-1.01] | 0.18 |
| Plasma urea | 1.06 [1.04-1.08] | <0.0005 | Plasma urea | 1.07 [1.04-1.09] | <0.0005 |
| Plasma creatinine | 1.01 [1.00-1.01] | <0.0005 | Plasma creatinine | 1.01 [1.00-1.01] | <0.0005 |
| Plasma sodium | 1.00 [0.98-1.02] | 0.87 | Plasma sodium | 0.99 [0.96-1.02] | 0.57 |
| Plasma haemoglobin | 0.98 [0.98-0.99] | 0.001 | Plasma haemoglobin | 0.98 [0.98-0.99] | <0.0005 |
| Natriuretic peptide levels | 1.47 [1.25-1.72] | <0.0005 | Natriuretic peptide levels | 1.77 [1.43-2.19] | <0.0005 |
| Plasma Troponin | 1.15 [0.99-1.33] | 0.07 | Plasma Troponin | 1.33 [1.11-1.59] | 0.002 |
| Plasma PENK | 1.57 [1.37-1.78] | <0.0005 | Plasma PENK | 1.71 [1.46-2.00] | <0.0005 |
